# Supplementary figures and images for: Plasticity of adult coralline algae to prolonged increased temperature and pCO2 exposure but reduced survival in their first generation
Source: PLoS One. 2020 Jun 23;15(6):e0235125. doi: 10.1371/journal.pone.0235125 (PMC7310705; doi:10.1371/journal.pone.0235125)

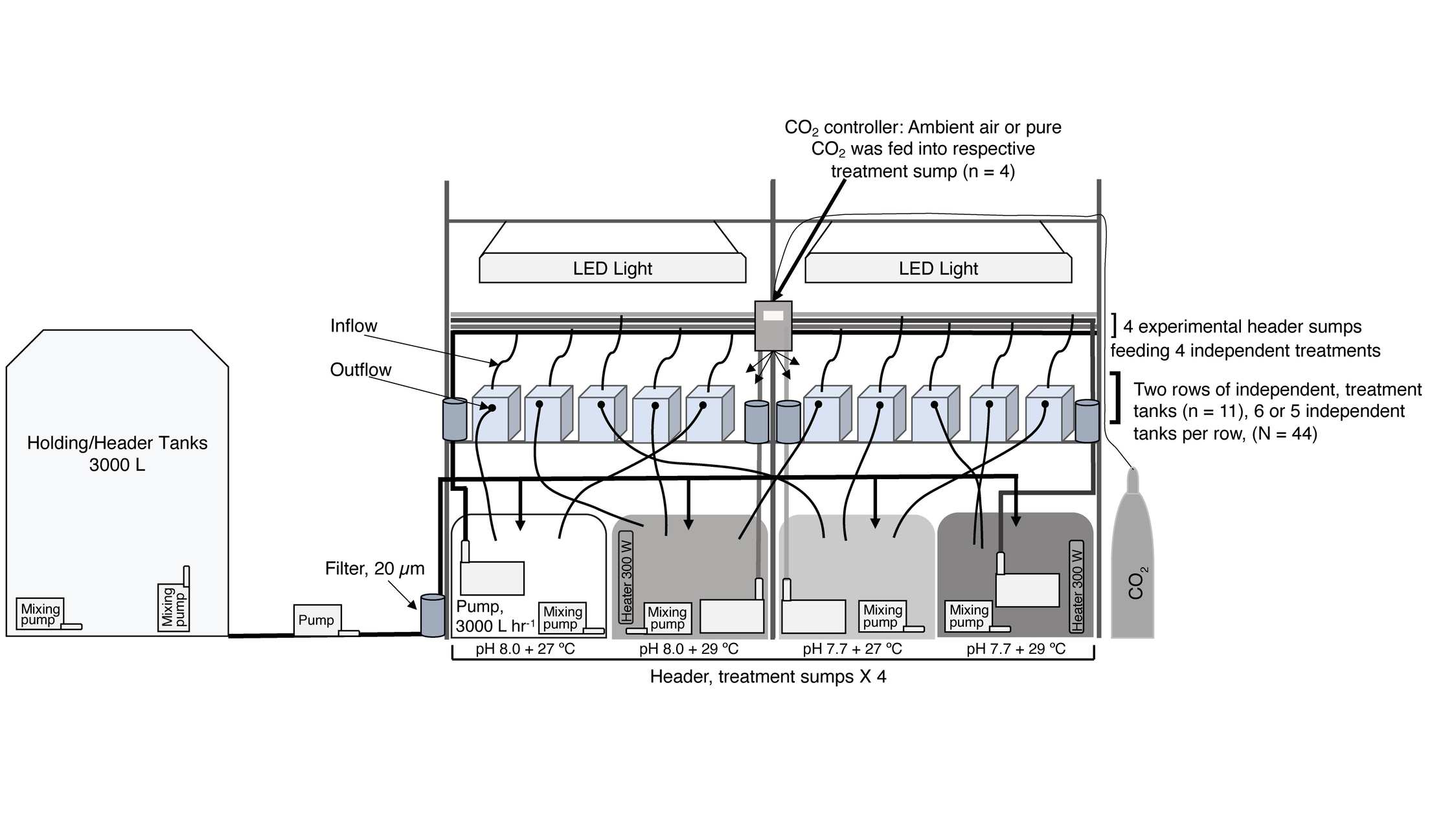

Supplement: S1 Fig — Diagram shows basic experimental setup including primary holding tank, treatment header sumps (n = 4), and independent treatment tanks (11 tanks per treatment). (TIF) [file pone.0235125.s001.tif]

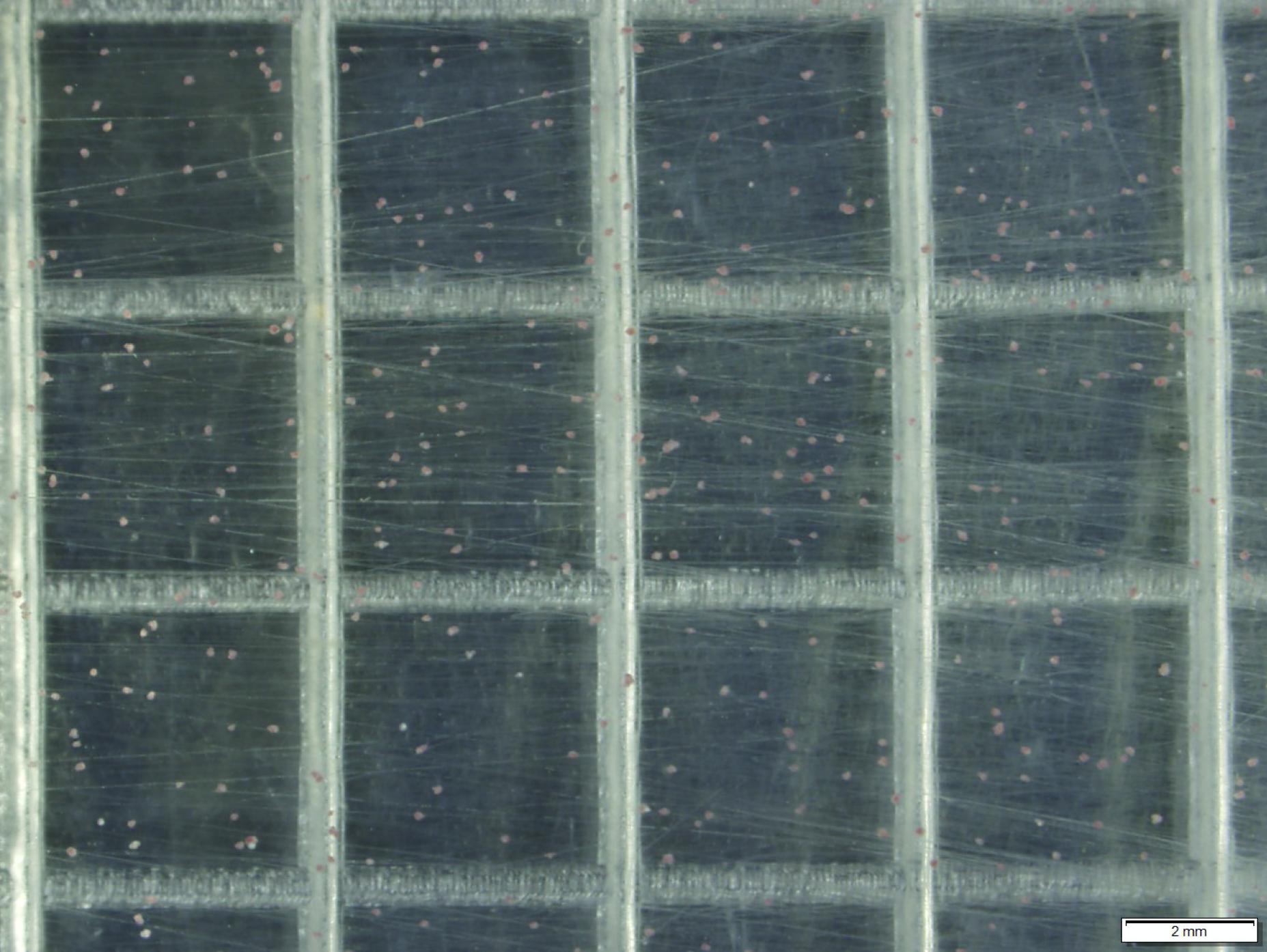

Supplement: S2 Fig — (TIF) [file pone.0235125.s002.tif]

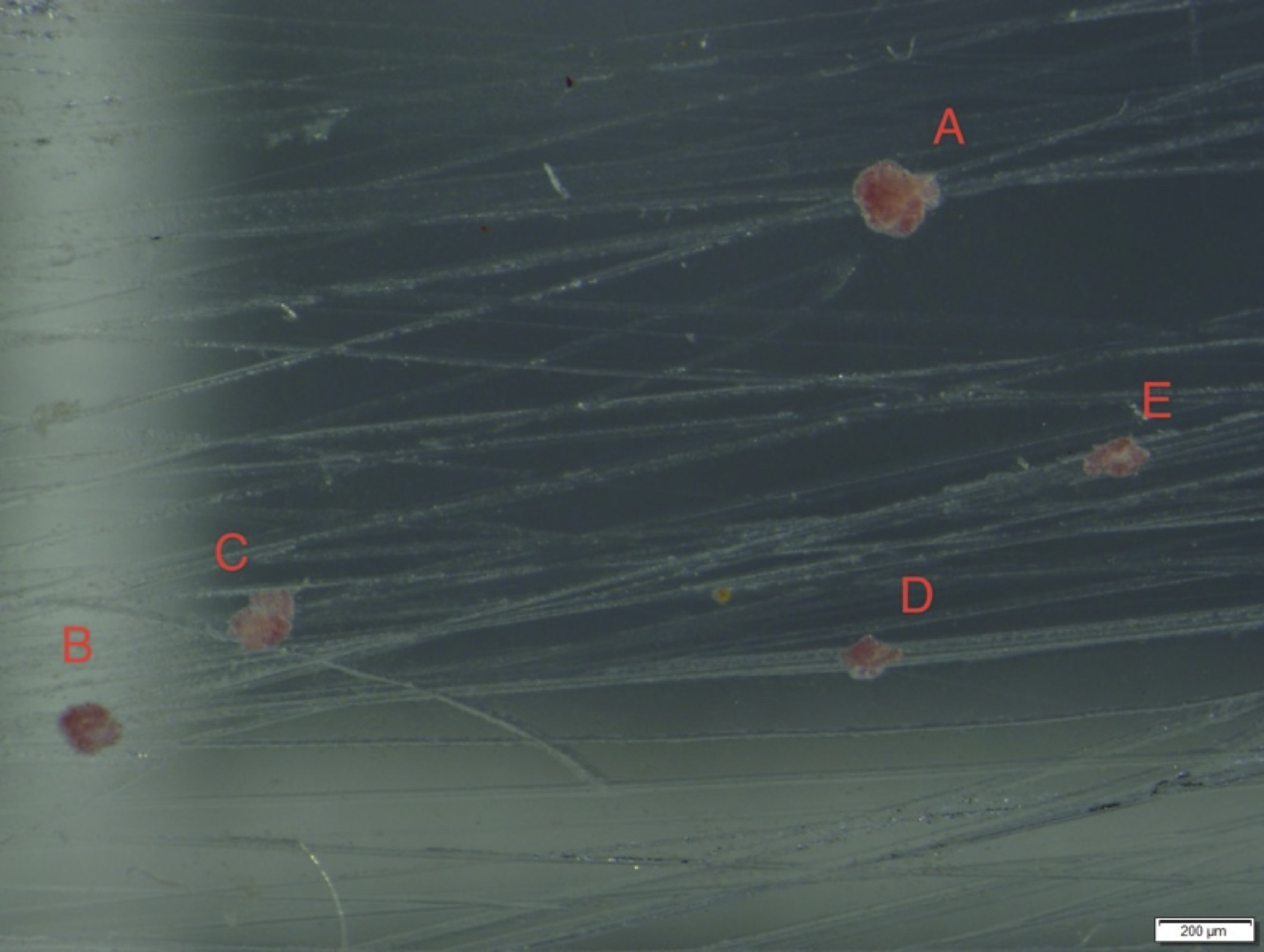

Supplement: S3 Fig — Letters identify individuals that were tracked throughout the experiment for survival and growth. (TIF) [file pone.0235125.s003.tif]
