# Supplementary material for: Plasticity of adult coralline algae to prolonged increased temperature and pCO2 exposure but reduced survival in their first generation
Source: PLoS One. 2020 Jun 23;15(6):e0235125. doi: 10.1371/journal.pone.0235125 (PMC7310705; doi:10.1371/journal.pone.0235125)
Supplement: S1 Table — Asterisk indicates parameters that were calculated using R package seacarb (v 3.2.12). High Mg-calcite was calculated for 16.4% calcite following methods from Diaz-Pulido et al. [16]. (PDF) [file pone.0235125.s004.pdf]

Treatment	pH 8.0 + 27 ºC	pH 8.0 + 29 ºC	pH 7.7 + 27 ºC	pH 7.7 + 29 ºC
Salinity (n = 150)	35.55 ± 0.03	35.61 ± 0.03	35.60 ± 0.04	35.67 ± 0.03
Temperature (ºC) (n = 150)	27.10 ± 0.02	29.10 ± 0.03	27.10 ± 0.03	29.10 ± 0.03
pH_T (n = 209)	8.02 ± 0.002	8.01 ± 0.002	7.70 ± 0.002	7.70 ± 0.002
Alkalinity (μequiv kg^{-1}) (n = 25)	2391.64 ± 4.10	2378.32 ± 3.76	2372.88 ± 6.22	2371.52 ± 4.72
DIC*	2077.13 ± 3.87	2043.27 ± 3.74	2228.46 ± 6.03	2199.86 ± 4.65
pCO_2 (μatm)*	443.73 ± 2.83	1039.92 ± 2.73	441.91 ± 5.09	1035.23 ± 6.04
[HCO^{3-}] (μmol kg^{-1})*	1838.80 ± 3.79	1795.90 ± 3.78	2076.97 ± 5.70	2042.61 ± 4.45
[CO_3^{-2}] (μmol kg^{-1})*	226.43 ± 0.95	236.08 ± 0.88	123.65 ± 0.54	130.84 ± 0.57
Ω_{cal}*	5.45 ± 0.02	5.7 ± 0.02	2.97 ± 0.01	3.16 ± 0.01
$\Omega_{High\ Mg\text{-}calcite}$	1.32 ± 0.02	1.42 ± 0.02	0.72 ± 0.03	0.79 ± 0.01
