# Supplementary material for: Plasticity of adult coralline algae to prolonged increased temperature and pCO2 exposure but reduced survival in their first generation
Source: PLoS One. 2020 Jun 23;15(6):e0235125. doi: 10.1371/journal.pone.0235125 (PMC7310705; doi:10.1371/journal.pone.0235125)
Supplement: S2 Table — Counts of spores were taken from an n = 5–6 adults. One outlier was removed from the increased temperature treatment, which was identified using Tukey’s rule and was more than 1.5 X the interquartile range. (PDF) [file pone.0235125.s005.pdf]

Source of variation	df	MS	*F*	p
Temperature	1	105648	1.614	0.211
pCO_2	1	194559	0.876	0.362
Temperature * pCO_2	1	36886	0.306	0.587
Residuals	17	120569		
